# Novel Method for the Synthesis of Hydroxycobalamin[*c*-lactam] and Its Impact on Melanoma Cells In Vitro

**DOI:** 10.3390/ijms26041540

**Published:** 2025-02-12

**Authors:** Zuzanna Rzepka, Magdalena Janus, Krzysztof Marciniec, Jakub Rok, Dorota Wrześniok

**Affiliations:** 1 Department of Pharmaceutical Chemistry, Faculty of Pharmaceutical Sciences in Sosnowiec, Medical University of Silesia in Katowice, 4 Jagiellońska, 41-200 Sosnowiec, Poland; magdajanus99@interia.pl (M.J.); chemlek@sum.edu.pl (J.R.); 2 Department of Organic Chemistry, Faculty of Pharmaceutical Sciences in Sosnowiec, Medical University of Silesia in Katowice, 4 Jagiellońska, 41-200 Sosnowiec, Poland; kmarciniec@sum.edu.pl

**Keywords:** cobalamin, antivitamin B12, hydroxycobalamin[*c*-lactam], melanoma, cobalamin deficiency

## Abstract

The ability to over-proliferate is a hallmark of cancer cells, so inhibiting proliferation is crucial for successful cancer treatment. Vitamin B12 (cobalamin) is among the factors necessary for replication of genetic material and cell division. There is currently no cobalamin antagonist with therapeutic use. Nevertheless, the idea of inhibiting cobalamin-dependent metabolic pathways as a potential anticancer strategy is of interest to many researchers. In this study, we investigated, for the first time, the impact of cobalamin deficiency on melanoma cells’ growth. To achieve a cobalamin-deficient state in cellulo, hydroxycobalamin[*c*-lactam] was used as an antivitamin B12. Here, we describe a new and efficient method for synthesizing this analog from hydroxycobalamin. Interestingly, no cytostatic effect of cobalamin deficiency was observed on C32 and COLO 829 melanoma cell lines. However, we show the variously enhanced pro-proliferative action of vitamin B12 towards these cells. The presented experimental model can be used for further studies on the effects of the cobalamin status on melanoma cells.

## 1. Introduction

Melanoma has been a huge public health problem for years, especially among the populations of highly developed countries. The United States has seen an increase in incidence of about 320% over the past 40 years, and the cancer is the fifth most common [1]. According to estimates, in 2040, the number of newly diagnosed melanoma patients will be approximately 510,000 globally, almost twice as many as in 2020, and the number of deaths from the disease could be as high as 96,000 [2].

In recent years, treatment options for patients with metastatic melanoma have been revolutionized by FDA approval of new therapeutic strategies, including a monotherapy with BRAF kinase inhibitors, combination therapy with MEK kinase inhibitors, immunotherapy with anti-CTLA-4 and anti-PD-L1 and PD-1 antibodies, as well as methods of stimulating the immune system with oncolytic viruses and adoptive immunotherapy [3,4,5]. Despite significant advances in the context of therapeutic solutions, cases of melanoma resistant to the available treatments are still encountered, due to the very rapid development of the disease, the early formation of distant metastases, and the effective genetic response of melanoma cells to external agents. Therefore, it is extremely important to conduct research exploring the properties of melanoma cells and develop innovative therapeutic strategies to achieve sustained melanoma remission [1,2,3,4,5,6].

A common feature of all types of cancer is the excessive cell proliferation ability, and thus the inhibition of growth is crucial for an effective treatment. One of the agents necessary for the synthesis of genetic material, and therefore cell division, is vitamin B12, also known as cobalamin (Cbl) [7]. The relationship between elevated cobalamin levels and solid tumors’ incidence was demonstrated for the first time in the late 1980s [8]. Hypercobalaminemia has also been reported in patients with hematologic and lymphoid malignancies [9,10,11]. Despite the theoretical rationale for using antivitamins B12 as antiproliferative agents, and despite the approval of folic acid (vitamin B9) antagonists, so far, no antivitamin B12 has been approved as a drug [7].

Cobalamin is a complex compound with a cobalt atom at the coordination center. The backbone of the molecule is a macrocyclic corin system made up of four pyrrole rings. The cobalt in the cobalamin molecule is linked to the nitrogen atoms of the pyrrole rings, the nitrogen atom of the 5,6-dimethylbenzimidazole riboside, and the ligand. Depending on the form of vitamin B12, the ligand can be a cyanide, methyl, 5′-deoxyadenosyl, hydroxyl group, or water molecule [12,13,14]. The metabolically active forms of vitamin B12 are methylcobalamin (Me-Cbl) and 5′-deoxyadenosylcobalamin, also known as adenosylcobalamin (Ado-Cbl). Hydroxycobalamin (OH-Cbl) and aquacobalamin are intermediate forms that are transformed in cells to Me-Cbl and Ado-Cbl. The synthetic derivative—cyanocobalamin (CN-Cbl)—is the most common form of vitamin B12 in pharmaceutical preparations due to its chemical stability and low cost. The intracellular reductive decyanation of CN-Cbl is a prerequisite for its conversion to the active forms: Me-Cbl and Ado-Cbl [15,16].

Cbl is involved in many biochemical processes, such as the metabolism of carbohydrate and lipids, the synthesis of myelin sheaths of nerve fibers, and the synthesis and methylation of DNA [17,18,19]. B12 in the form of Me-Cbl is a cofactor for methionine synthase (EC 2.1.1.13), which transfers the methyl group from 5-methyltetrahydrofolate (5-methylTHF) to homocysteine (HCY), leading to the formation of methionine and tetrahydrofolate (THF) [20,21]. THF is then converted to 5,10-methylenetetrahydrofolate, which is necessary for thymidine nucleotide synthesis. Cobalamin deficiency causes a blockage of these transformations, leading to the accumulation of HCY and 5-methylTHF and the inhibition of DNA synthesis [22,23]. Ado-Cbl plays a key role in cellular metabolism as a cofactor of methylmalonyl-CoA mutase (EC 5.4.99.2), which catalyzes the isomerization of methylmalonyl-CoA to succinyl-CoA. This reaction funnels the products of propionate metabolism (generated from the breakdown of branched-chain amino acids, odd-chain fatty acids, and cholesterol) into the tricarboxylic acid cycle [16].

Considering the role of cobalamin in rapidly dividing cells, we decided to investigate whether cobalamin deficiency can affect melanoma cells’ growth. To investigate this, we cultured melanoma cell lines C32 and COLO 829 in hypocobalaminemic conditions. This was achieved using medium supplemented with hydroxycobalamin[*c*-lactam] (OH Cbl[*c*-lactam]), a B-ring analog of B12. This compound structurally resembles Cbl, but it is incapable of performing vitamin B12’s function and therefore blocks cobalamin-dependent transformations [7,24,25]. In this study, a model of cobalamin deficiency in melanoma cells was developed and analyzed for the first time. In addition, in the present article, we describe the new method for the synthesis of OH-Cbl[*c*-lactam], which can successfully be used as an inducer of cobalamin deficiency in various experimental models.

## 2. Results

### 2.1. A New Method for the Synthesis of OH-Cbl[c-Lactam]

OH-Cbl[*c*-lactam] was prepared from commercially available OH-Cbl hydrochloride, adapting the CN-Cbl[*c*-lactam] synthesis procedure developed by Bonnet et al. [26].

**Scheme 1:**
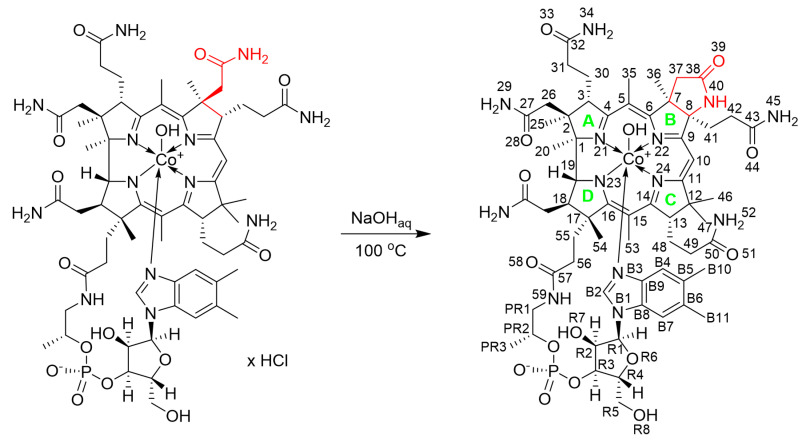
Synthesis of hydroxycobalamin[c-lactam] from hydroxycobalamin hydrochloride.

The cyclization reaction of the c chain in the hydroxocobalamin system was carried out in an alkaline substrate solution at the boiling temperature while simultaneously passing a stream of air through the mixture (Scheme 1). The reaction product was isolated from the mixture by extraction. The primary phenol extraction procedure separates the water-soluble cobalamin salts. However, extraction of aqueous solutions with phenol poses problems due to the formation of emulsions. Therefore, in our work, we used a phenol solution in methylene chloride as the organic phase in the extraction [27]. 

**Figure 1:**
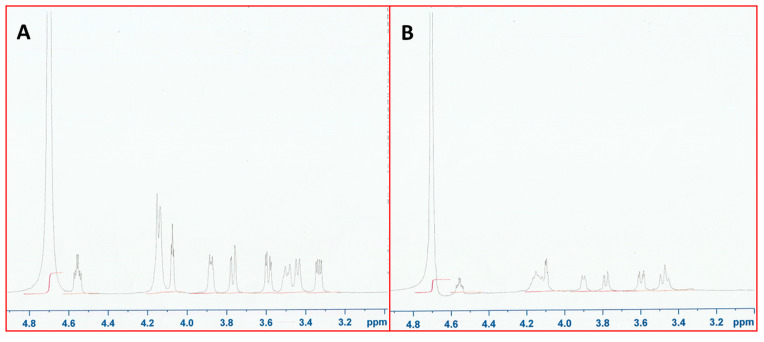
Aliphatic range of the ^1^H NMR spectrum of hydroxocobalamin (**A**) and hydroxocobalamin[*c*-lactam] (**B**).

The structure of lactam was confirmed using ^1^H NMR and ^13^C NMR spectra (Figures S1 and S2) and was consistent with the data in the literature [28,29,30]. ^13^C NMR spectroscopy plays a key role in confirming the structure of the obtained lactam [30]. According to the data from the literature for cyanocobalamin and its *c*-lactam, the signals of the C38 and C8 carbon atoms in lactam are shifted towards a downfield field, with a particularly strong revealing effect occurring in the case of the C8 atom. In cyanocobalamin, they resonate at δ = 175.13 and 55.72 ppm, respectively, and in its c-lactam at δ = 177.1 and 74.1 ppm. In the product we obtained, the carbon atoms mentioned resonated at 176.1 and 74.1 ppm, respectively. Moreover, the ^1^H NMR spectrum shows the lack of the H8 proton signal present in the substrate at 3.33 ppm (Figure 1).

HR MS analysis was also performed (Figure S3). As expected, no molecular peak of the product was distinguished in the spectrum obtained with an ESI source in the positive ion mode. The main peak found was a peak with a mass of 663.7759 Da, corresponding to a doubly charged fragment ion resulting from the loss of a water molecule by the analyzed substance [M+2H^+^-H_2_O]^2+^ [31].

We obtained the compound with a yield of 31.9% and an HPLC purity of 95% (Figure S4).

### 2.2. The Effect of OH-Cbl[c-Lactam] on Melanoma Cells In Vitro

#### 2.2.1. The Impact of OH-Cbl[c-Lactam] on the Extracellular Homocysteine Level of Melanoma Cells

**Figure 2:**
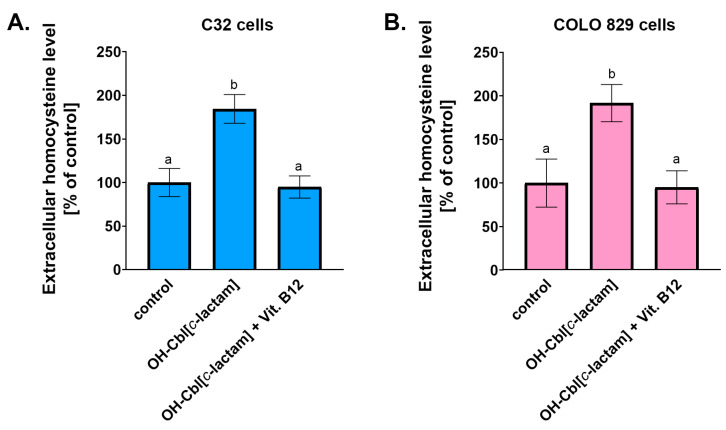
The effect of OH-Cbl[*c*-lactam] on the homocysteine concentration in the culture medium of C32 (**A**) and COLO 829 (**B**) cells. The cells were cultured for 14 days with OH-Cbl[*c*-lactam] at a concentration of 100 µg/mL alone or in combination with vitamin B12 (vit. B12) at the same concentration. Data were normalized to the cell number and presented as a % of the untreated control. Bar chart representing the mean ± SD (standard deviation) of 3 independent experiments in at least triplicate. Different letters above the bars indicate a statistically significant difference at *p* < 0.05.

Homocysteine is a well-known biomarker of hypocobalaminemia. This is because functional cobalamin deficiency leads to inhibition of methionine synthase, resulting in the accumulation of HCY in cells [20,21,25]. Under in vitro conditions, the excess of HCY is excreted into the culture medium [32,33]. In our study, we determined the level of HCY in the culture medium of tested melanoma cells to detect cobalamin deficiency. Medium samples were collected on the 14th day of the culture. Due to the practical aspects of the experimental model, we considered a time of 2 weeks to be optimal (a longer culture time could be inconvenient, problematic, and cost-consuming for future studies). The choice of concentration of B12 antivitamin resulted from our preliminary experiments: C32 and COLO 829 cells were cultured in the presence of OH-Cbl[*c*-lactam] at a concentration of 50 µg/mL for 14 days. During passage, we analyzed the cell count and viability and observed the cells under the microscope. We did not observe any differences compared to the control. We therefore repeated the experiment but with twice the concentration of the antivitamin. At a concentration of 100 µg/mL, we also observed no change in melanoma cell proliferation or survival. Nevertheless, we quantified the marker of hypocobalaminemia, i.e., homocysteine. The ELISA results showed that both C32 and COLO 829 cells cultured with OH-Cbl[*c*-lactam] secreted significant amounts of HCY (Figure 2)—the concentrations were approx. 180% of the control values. The extracellular level of HCY in the population of cells cultured simultaneously with OH-Cbl[*c*-lactam] and vitamin B12 was the same as that of the untreated control, suggesting an antagonistic effect of the test substances on methionine synthase.

#### 2.2.2. Effect of Vitamin B12 and Its Antagonist on Melanoma Cells’ Proliferation

**Figure 3:**
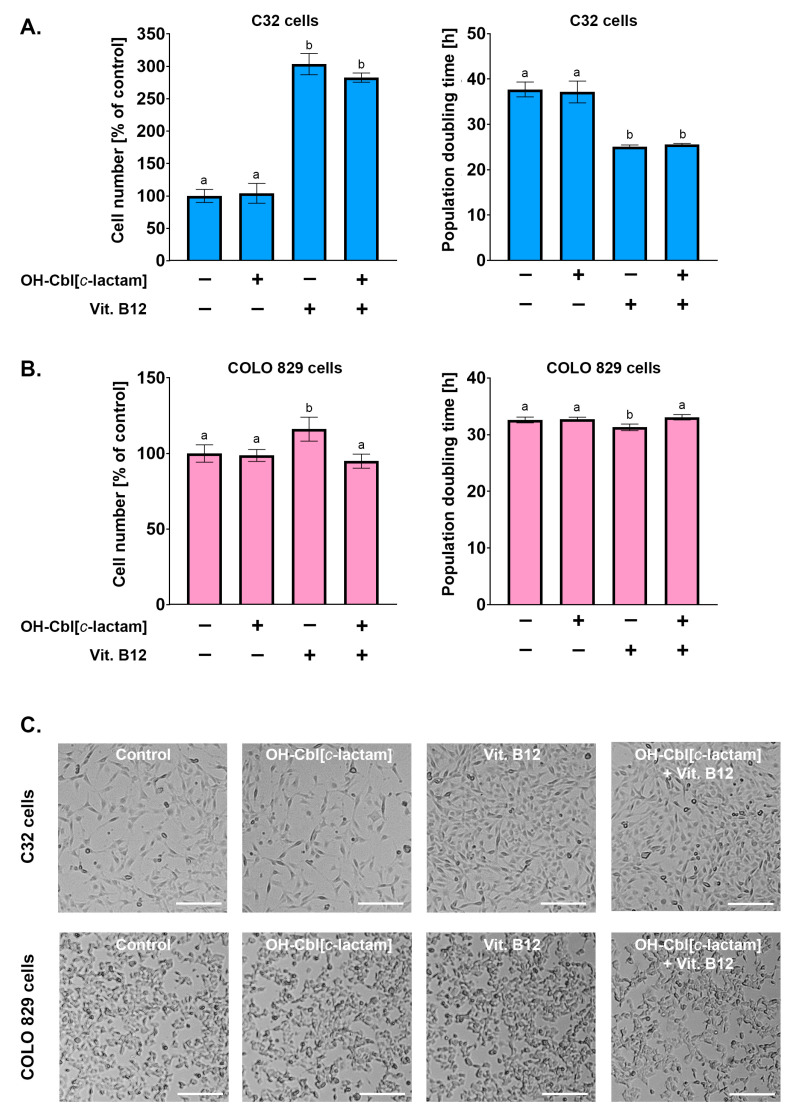
The effect of the cobalamin status on the proliferation of melanoma cells. The relative cell number and population doubling time (PDT) of melanoma cell lines C32 (**A**) and COLO 829 (**B**) are presented. The cells were cultured for 14 days with OH-Cbl[*c*-lactam] at a concentration of 100 µg/mL or/and vitamin B12 at a concentration of 100 µg/mL. Bars represent the mean ± SD (standard deviation) of 3 independent experiments in at least triplicate. Different letters above the bars indicate a statistically significant difference at *p* < 0.05. Melanoma cells were observed under an Eclipse TS-100-F inverted microscope (Nikon, Tokyo, Japan), and representative images are shown (**C**), where the scale bar = 100 µm.

After 14 days of culturing melanoma cells in the presence of OH-Cbl[*c*-lactam], when the level of HCY in the medium indicated a functional cobalamin-deficient state, the cell count were assessed by an image cytometer. The population doubling time (PDT) was calculated based on the results, as described in the methodology section. The results for the C32 and COLO 829 cell lines are shown in Figure 3A and Figure 3B, respectively.

The proliferation of C32 cells treated with vitamin B12 at a concentration of 100 µg/mL was highly accelerated—a ca. 3-fold higher cell number than in the control and an estimated PDT that reduced from 38 h to 25 h. The addition of a cobalamin antagonist did not reduce the vitamin B12-induced effect. There were no differences in proliferation rates between controls and cells cultured in the presence of OH-Cbl[*c*-lactam] alone.

The results obtained for COLO 829 cells showed a different response to the vitamin B12 status compared to the C32 line. When cultured in the presence of vitamin B12, the proliferation of COLO 829 cells increased by about 16% compared to the control (a small but statistically significant increase, *p*-value 0.0369). Compared to the control, the PDT of cells treated with vitamin B12 decreased only slightly (from 32.5 h to 31 h), although the difference was statistically significant. The cobalamin antagonist reduced the observed effect of vitamin B12 on the proliferation rate of COLO 829 cells; however, it did not affect cell proliferation on its own.

The microscopic images shown in Figure 3C are consistent with the cell count results.

#### 2.2.3. Cell Cycle Distribution of Melanoma Cells Treated with Vitamin B12 and Its Antagonist

**Figure 4:**
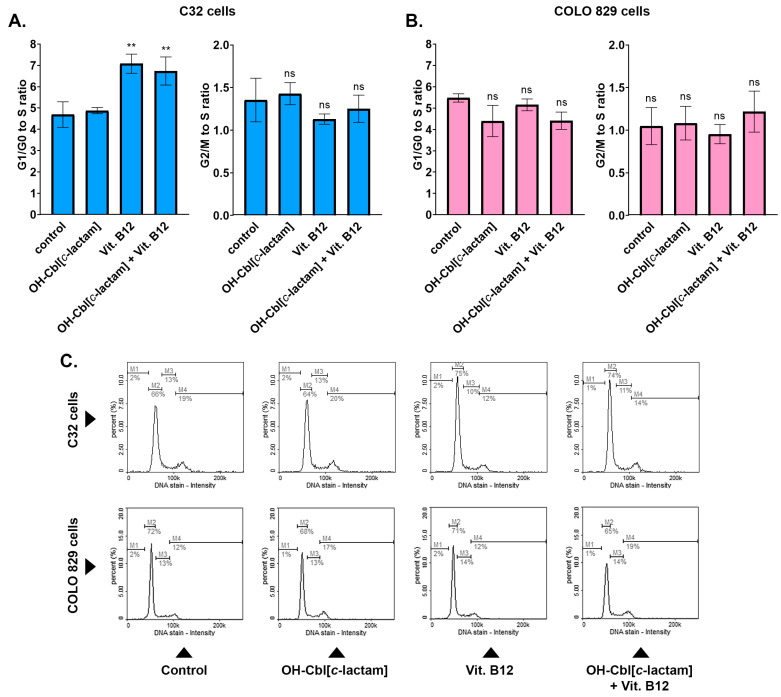
The impact of vitamin B12 and its antagonist on the cell cycle distribution of human melanoma cells C32 (**A**) and COLO 829 (**B**). The cells were cultured for 14 days with OH-Cbl[*c*-lactam] at a concentration of 100 µg/mL or/and with vitamin B12 (vit. B12) at the same concentration. The G1/G0-to-S ratio and G2/M-to-S ratio were calculated. Bars represent the mean ± SD (standard deviation) of 3 independent experiments in at least triplicate, ** *p* < 0.01, ns = not significant. Representative histograms from the analysis (**C**) show the populations of cells in the sub-G1(M1), G1/G0 (M2), S (M3), and G2/M (M4) phases.

Figure 4 shows the results of cytometric cell cycle analysis. We calculated the G1/G0-to-S ratio and G2/M-to-S ratio to determine whether cell cycle arrest reached significance at the G1 or G2/M checkpoint. C32 melanoma cells when cultured for 14 days in the presence of cobalamin showed accumulation in the G1/G0 phase (Figure 4A). OH-Cbl[*c*-lactam] alone did not affect C32 cell cycle progression. The compound, when given concurrently with vitamin B12, did not reduce the effect of the vitamin. Based on the analysis of the calculated ratios, no significant changes in the cell cycle were observed for COLO 829 cells under any of the conditions tested (Figure 4B). No increase in the percentage of cells in the sub-G1 phase was detected in any of the samples analyzed.

## 3. Discussion

As a cofactor of the enzymes methionine synthase and methylmalonyl-CoA mutase, cobalamin is involved in nucleic acid synthesis and energy metabolism [17,18]. Consequently, cells with accelerated proliferation and metabolism may be particularly sensitive to cobalamin deficiency. These features are characteristic of cancer cells, suggesting that cobalamin deficiency may be detrimental to them [7,34]. This was confirmed by studies on human leukemia cell line HL60 [35], human glioblastoma cell line U87-MG [24], and human liver cancer cell line HepG2 [36]. In addition, increased expression of the proteins responsible for cobalamin transport (transcobalamin I, transcobalamin II, and transcobalamin II receptor) in cancer cells was found to be associated with resistance to chemotherapy and aggressive tumor growth [37,38]. Vitamin B12 plays a key role in the methionine cycle. Scientific evidence indicates that most cancer cells show high methionine cycle activity and are dependent on methionine for continued growth [39]. Miousse et al. [40] revealed that methionine deprivation potentiated the effects of radiotherapy in a mouse melanoma model via the *Mat2a* pathway and diminished the melanoma metastatic potential.

Considering the above arguments, the concept of inducing vitamin B12 deficiency in cancer cells is interesting in the context of basic cancer research and potential anticancer strategies. Inducing a hypocobalaminemic state in vitro is not easy due to the presence of cobalamin in the culture media and in bovine serum, which is a standard additive to the culture media. Moreover, different cell types differ in their requirements for this vitamin, so there is no universal in vitro research model for hypocobalaminemia. Studies have been published in which antivitamins B12 have been used to induce cobalamin deficiency. For example, Mutti et al. [41] observed an increase in serum homocysteine and methylmalonic acid after treating mice with 4-ethylphenyl-cobalamin for 27 days. Stabler et al. [25] synthesized analogs: OH-cbl[*c*-lactam], a B–ring analog, as well as OH-Cbl[e-dimethylamide] and OH-Cbl[e-methylamide], two C-ring analogs, which have been shown to be effective inhibitors of vitamin B12-dependent enzymes in rats after 14-day treatment. Ruetz et al. [42] described the preparation of a potential antivitamin B12—phenylethynylcobalamin. Several years later, Ruetz et al. [43] published an article that included the synthesis of 2,4-difluorophenylethynylcobalamin (F2PhEtyCbl), which was presented as an improved analog, resistant to hydrolysis and capable of inhibiting CblC, a protein in humans responsible for the processing and intracellular transport of vitamin B12.

In this article, we have presented for the first time an experimental model of hypocobalaminemia in human melanoma cells (C32 and COLO 829 cell lines). We used hydroxycobalamin[*c*-lactam] to block cobalamin activity. The properties of this compound as a B12 antivitamin have previously been exploited by Stabler et al. [25] and Haegler et al. [36], as well as in our previous work [24,28,44]. The commercial availability of OH-Cbl[*c*-lactam] is limited, so we developed and described a method for the synthesis of this derivative.

**Scheme 2:**
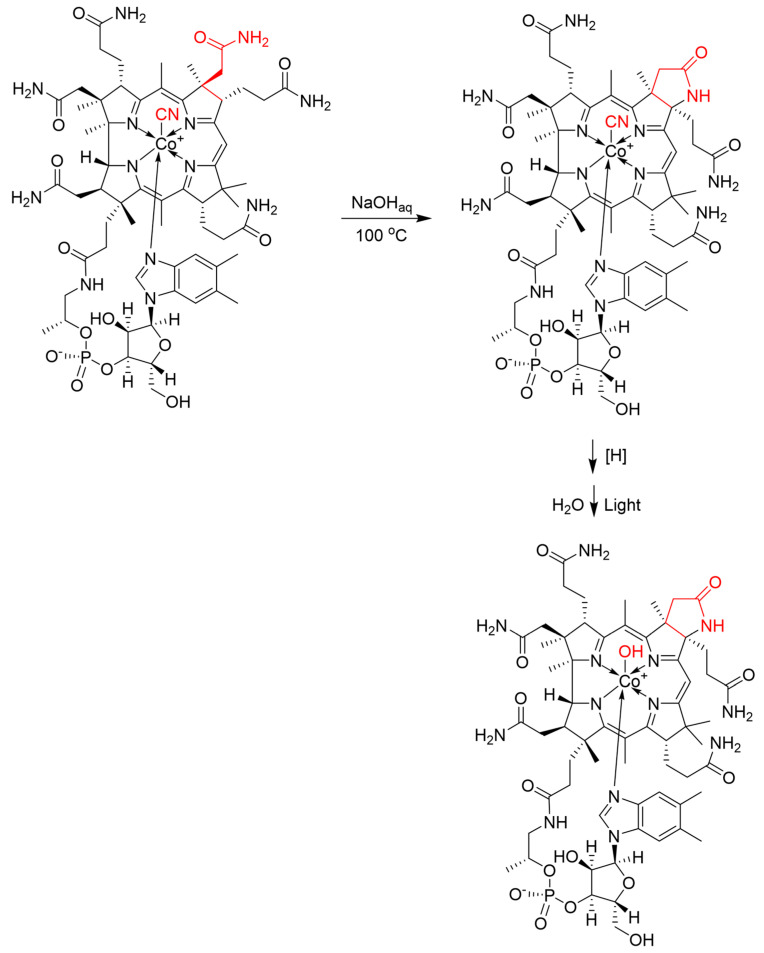
Previously reported synthesis of OH-Cbl[*c*-lactam].

Previous publications contain plentiful information regarding the synthesis of OH-Cbl[*c*-lactam] and its derivatives. However, all these syntheses are based on the synthetic path presented in Scheme 2. In this method, the substrate is CN-Cbl, which is initially subjected to a lactamization reaction. In the classic work by Bonnett et al. from 1957 [26], the authors obtained both vitamin B12 lactam and lactone. Lactam was obtained by heating cyanocobalamin in a diluted aqueous NaOH solution (yield of 51%), while lactone was obtained by reacting cyanocobalamin with chloramine T. Although many modifications of this method for obtaining lactone were published in subsequent years (e.g., using NBS or the NCS/NaI system), the Bonnett method for obtaining cyanocobalamin lactam is still widely used [27]. This is probably due to the fact that this method is very simple to perform, because it takes place in an aqueous solution and uses commonly available and cheap reagents, which is currently in line with the principles of green chemistry. The next stage of the synthesis presented in Scheme 2 requires the reduction of the central cobalt(III) ion to cobalt(II). The most commonly used reducing reagents are NaBH_4_, Zn, sodium formate, chromium(II) acetate, and Na_2_SO_3_ [13,28]. Electrochemical methods are also efficient. Reduced cobalt(II) ions are very reactive and spontaneously oxidize to cobalt(III) ions. Moreover, under the influence of visible light (formerly fluorescent lamps, now LEDs), OH-Cbl[*c*-lactam] is obtained. Both these steps, the reduction and replacement of the cyanide group with the hydroxyl group, proceeded with moderate yields, meaning the total yield of hydroxycobalamin *c*-lactam did not exceed several percent.

In our method, we used hydroxycobalmin (in the form of hydrochloride) as a substrate, which was different from the original procedure of Bonnet and colleagues [26]. We subjected this compound to a lactamization reaction under the conditions developed by Bonnett et al., obtaining the title compound in one step (with a total yield of 32%). Given the many advantages of this method, we did not make any changes to the procedure. However, carrying out the reaction in one stage resulted in a high overall synthesis yield, which should be considered an important result. It should be emphasized that the original, three-step method requires larger amounts of reagents and solvents and often specialized equipment (as in the case of electrochemical reduction). The workload is also greater, especially when the need to purify the products of each reaction step is also taken into account.

We have described a cobalamin deficiency model involving a long-term culture with OH-Cbl[*c*-lactam] for normal human melanocytes (10 μg/mL of OH-Cbl[*c*-lactam] for 24 days) [28], normal human astrocytes (20 μg/mL, 27 days) [42], and human U87-MG glioblastoma multiforme cells (50 µg/mL, 18 days) [24]. In the current study, based on our preliminary experiments, we cultured melanoma cells C32 and COLO 829 for 14 days with OH-Cbl[*c*-lactam] at a concentration of 100 ug/mL. The qualitative analysis of homocysteine in culture medium confirmed that a functional cobalamin deficiency was obtained in the tested melanoma cells: homocysteine ejection into the medium was observed in the cell population cultured with OH-Cbl[*c*-lactam], while simultaneous addition of vitamin B12 at the same concentration reduced this effect. The use of other structural analogs of vitamin B12 with potential properties of an antivitamin B12 (such as the analogs described by Zelder et al. [7]) will be an interesting topic for future research, to further optimize the experimental model.

Surprisingly, our results showed that cobalamin deficiency in melanoma cells did not cause an inhibition of proliferation. This was different in the case of glioblastoma U87-MG cells, where a 160% increase in homocysteine levels relative to controls was associated with a decrease in proliferation of more than 70% [24]. The lack of an antiproliferative effect in cobalamin-deficient melanoma cells may be due to compensatory mechanisms, e.g., an upregulated serine biosynthetic pathway and enhanced role of serine/glycine metabolism in de novo nucleotide biosynthesis. We suggest that the protein that may be relevant here is serine hydroxymethyltransferase (SHMT). SHMT overexpression and its role in promoting cell proliferation has been shown in various human tumors [45]. SHMT catalyzes the reversible conversion of serine to glycine with the transfer of the one-carbon unit from tetrahydrofolate to 5,10-methylenetetrahydrofolate (CH_2_-THF). CH_2_-THF is used in thymidine synthesis and is a precursor to other folate species required for purine biosynthesis [46,47]. Investigating the activity/level of SHMT in melanoma cells in a cobalamin-deficient model will therefore be of interest for further research. In addition, it was found that the *PHGDH* gene, encoding the rate-limiting enzyme of the serine synthesis pathway, is frequently amplified in melanoma cells, suggesting that serine metabolic reprogramming is crucial for the progression and drug resistance of melanoma [48,49].

As for glioblastoma cells [24], in the current study, we observed a significant increase in the proliferation of melanoma cells cultured with vitamin B12. This increase was particularly high for the C32 melanoma cell line. The obtained results suggest a significant role of cobalamin in the growth of these tumors. It is worth mentioning that there is no clear clinical evidence demonstrating a direct effect of vitamin B12 on human cancer development [50]. Nevertheless, there are reports that cancer patients with elevated cobalamin levels had higher mortality than those with the normal levels [9,51,52]. In COLO 829 cells, the increase in the proliferation rate induced by vitamin B12 was lower and the addition of the antagonist reduced the proliferation dynamics to the control level. We drew similar conclusions previously for glioblastoma cells [24]. Interestingly, in the case of C32 cells, the addition of the cobalamin antagonist did not reduce the hyperproliferation induced by vitamin B12, which may be due to the extremely strong pro-proliferative effect of cobalamin relative to C32 cells. We suggest that the different responses to vitamin B12 between the C32 and COLO 829 lines may be due to a difference in the expression of a plasma membrane receptor TCblR (encoded by the *CD320* gene [53]), which is required by cells for the uptake of vitamin B12. However, this hypothesis requires further molecular studies.

It is known that different types of normal cells differ in their sensitivity to the vitamin B12 status. It depends on their function, rate of division, or ability to store vitamin B12. For example, bone marrow cells or cells of the nervous system show marked changes in homeostasis in a cobalamin-deficient state, which manifests clinically as symptoms of vitamin B12 deficiency [12,17]. Hence, it is hypothesized that cancer cells of different origins may differ in their sensitivity to cobalamin deficiency. The current work on melanoma cells and our previous work on U87-MG cells suggest that different types of cancers may respond differently to the vitamin B12 status.

Cytometric cell cycle analysis showed the absence of apoptotic cells (in the sub-G1 phase) in the melanoma cell populations treated with cobalamin and its antagonist. An increase in the percentage of cells in the G1/G0 phase was observed in vitamin B12-treated cells compared to the controls. The arrest in the G1 phase may be due to the high density of cells in this sample, as shown in the study by Hannan et al. [54]. Functional cobalamin deficiency, through disruption of cellular DNA synthesis, can block the cell’s transition from the G2 phase to mitosis. Such an effect was shown previously [24] for U-87 MG glioblastoma cells treated with the same cobalamin antagonist.

## 4. Conclusions

In conclusion, in this article, we have described a new method for the synthesis of hydroxycobalamin[*c*-lactam] and demonstrated that the obtained compound can be used to induce cobalamin deficiency in melanoma cells. Nevertheless, in the presented study using C32 and COLO 829 cell lines, no antiproliferative or cytotoxic effect of cobalamin deficiency was observed. We showed the pro-proliferative effect of vitamin B12, which was particularly enhanced in the C32 cell line. The presented experimental model can be used for further studies concerning cobalamin deficiency in melanoma cells, e.g., studies on the molecular mechanisms with which these cells are able to proliferate despite a vitamin B12 depletion. Investigating these mechanisms and methods to counteract them may provide a basis for the development of new anticancer strategies based on blocking vitamin B12 metabolism or transport.

## 5. Materials and Methods

### 5.1. Chemistry

#### 5.1.1. General Chemistry Methods

Analytical standard hydroxocobalamin hydrochloride was purchased from Sigma Aldrich (St. Louis, MO, USA). All other commercially available reagents were of the highest purity (from Sigma-Aldrich, Fluorochem (Glossop, UK), and AlfaAesar (Haverhill, MA, USA). NMR spectra (^1^H, ^13^C) were recorded on a Bruker Ascend 600 (Bruker Corporation, Billerica, MA, USA) and were reported in ppm using a D_2_O solvent for calibration. High-resolution mass spectra were recorded on a Bruker Impact II (Bruker Corporation, Billerica, MA, USA) equipped with an ESI source of ions. HPLC analysis was performed on a Dionex UltiMate 3000 kit (Thermo Fisher Scientific, Waltham, MA, USA). An Accucore RP-MS HPLC column (Thermo Fisher Scientific) was used with a length of 150 mm and a diameter of 2.1 mm. ACN/1% aqueous HCOOH 80/10 *v*/*v* solution was used as the eluent. The determinations were made at a wavelength of 365 nm [55].

#### 5.1.2. Synthesis of OH-Cbl[c-Lactam]

OH-Cbl hydrochloride (545 mg, 0.4 mmol) was dissolved in 100 mL of an aqueous solution of sodium hydroxide at a concentration of 0.1 mol/dm^3^ and heated at 100 °C for 10 min. At the same time, a strong stream of air passed through the solution. After being cooled to room temperature, the brown solution was acidified with 1% aqueous hydrochloric acid, and the red product was extracted. The extraction was performed with a solution of phenol in methylene chloride. The stock solution was obtained by dissolving phenol (100 g) in methylene chloride (100 mL). The post-reaction mixture was extracted with 5 × 20 mL of phenolic solution. The combined organic extracts were washed 2 times with 20 mL of distilled water. Then, 900 mL of methylene chloride was added to the organic layer. OH-Cbl[*c*-lactam] was extracted again from the organic layer with portions of distilled water (5 × 50 mL). The combined aqueous extracts were then washed with 3 × 100 mL of methylene chloride to remove traces of phenol. The red aqueous extract was then concentrated at 20 mL under reduced pressure in a water bath. The product was then applied to a column (20 × 1 cm) of Amberlite XAD-2 and eluted with 200 mL of distilled water. The aqueous eluate was concentrated at 10 mL and 150 mL acetone added and a faint turbidity was produced. The solution gradually deposited long red needles (172 mg, 31.9%) of OH-Cbl[*c*-lactam].

^1^H NMR (D_2_O) δ: 7.07 (s, HB2), 6.49 (s, HB7), 6.33 (s, HB4), 6.11-6.12 (m, HC10 and HR1), 4.55 (td, J = 8.4, J = 4.3 Hz, HCR3), 4.10–4.16 (m, HR4, HC19 and HC3), 3.89 (d, J = 12.9, H_a_ of HR5), 3.79 (d, J = 13.0, H_b_ of HR5), 3.59 (dt, J = 14.0, 2.3 Hz, Ha of HPR1), 3.45 (d, J = 10.3 Hz, HC13), 2.76–2.86 (m, HC18, HC41 and HPR1), 2.55–2.59 (m, HC55′ and H_3_C53), 2.46–2.50 (m, HC26), 2.32 (s, H_3_C35), 2.17 (s, HB10 and HB11), 2.15–2.16 (m, HC37 and HC42), 1.92–2.09 (m, HC30, and HC48), 1.82 (s, H_3_C25), 1.79–1.80 (m, HC55 and HC60), 1.41 (s, H_3_C47), 1.34 (s, H_3_C36), 1.23 (s, H_3_C54), 1.21 (s, HPR3), 1.11–1.12 (m, HC41 and H_3_C46), 0.43 (s, HC20); ^13^C-NMR (D_2_O) 177.9 (C50), 177.6 (C32), 177.3 (C11), 176.1 (C38), 176.0 (C16 and C43), 175.8 (C27 and C61), 175.5 (C4), 174.5 (C57), 167.1 (C9), 163.8 (C14 and C6), 142.4 (CB2), 137.7 (CB8), 133.9 (CB5), 131.8 (CB6), 129.3 (CB9), 116.8 (CB4), 111.9 (CB7), 107.5 (C5), 104.9 (C15), 90.8 (C10), 87.8 (CR1), 85.6 (C1), 81.7 (CR4), 75.6 (C19), 74.1 (C8), 72.7 (CR3 and CPR2), 68.5 (CR2), 60.1 (CR5), 58.9 (C17), 57.9 (C3), 53.2 (C13), 51.9 (C7), 47.6 (C12) 46.1 (C2), 45.2 (CPR1), 43.7 (C37), 41.5 (C26), 39.4 (C18), 34.9 (C31), 34.4 (C49), 32.3 (C55), 32.2 (C56), 31.3 (C60), 30.2 (C47), 29.5 (C41), 29.4 (C42), 27.9 (C48), 25.2 (C30), 20.0 (C20), 19.9 (C35), 19.7 (C46), 19.8 (CB10 and CB11), 18.9 (CPR3), 17.1 (C54), 16.6 (C35), 16.2 (C25), 15.4 (C53). HRMS (ESI): *m*/*z* 663.7759 (100, [C_62_H_87_CoN_13_O_15_P + 2H-H_2_O]^2+^, *m*/*z*_calc_: 663.7777.

Data from the compound’s identification are available as Supplementary Materials.

### 5.2. Cell Culture

This study was conducted using cultures of human amelanotic melanoma cell line C32 and melanotic melanoma cell line COLO 829, purchased from the ATCC (American Type Culture Collection, Manassas, VA, USA). The culture medium for C32 cells was Dulbecco’s Modified Eagle Medium—high glucose (Sigma Aldrich, Darmstadt, Germany) and COLO 829 were cultured in RPMI (Roswell Park Memorial Institute) 1640 medium (Sigma Aldrich, Darmstadt, Germany). The media were enriched with 10% fetal bovine serum (FBS Premium from PAN Biotech GmbH, Aidenbach, Germany), 10 μg/mL neomycin sulfate (Life Technologies Corporation, Grand Island, NY, USA), 0.25 μg/mL amphotericin (Sigma Aldrich Chemie GmbH, Steinheim, Germany), and 100 IU/mL penicillin G sodium salt (Sigma Aldrich Chemie GmbH, Steinheim, Germany). The cultures were grown in the Binder CB 160 incubator (BINDER GmbH, Tuttlingen, Germany), providing constant conditions of 37 °C and 5% CO_2_.

### 5.3. Treatment of Cells with OH-Cbl[c-Lactam]

We had four parallel 14-day cultures for each melanoma line: (1) in standard medium (control), (2) with OH-Cbl[*c*-lactam] at a 100 μg/mL concentration, (3) with vitamin B12 (Polfa Warszawa S.A., Warszawa, Poland) at a 100 μg/mL concentration, and (4) with a combination of these two substances. Each time, cells were seeded in T-25 flasks (50,000 cells per flask), and the medium was changed every 2/3 days. Cells were passaged when the confluence was about 80%.

### 5.4. Determination of the Population Doubling Time

Analysis of the cell count and viability was performed using a NucleoCounter NC-3000 fluorescence image cytometer (ChemoMetec, Lillerød, Denmark), combined with the NucleoView NC-3000 Software. Cells adhering to the bottom of the bottle were detached using trypsin-EDTA solution (Life Technologies Limited, Paisley, UK) and then resuspended in culture medium. The suspension was collected in a Via1-Cassette (ChemoMetec, Lillerød, Denmark), filled with acridine orange and DAPI (4′,6-diamidino-2-phenylindole). To calculate the population doubling time (PDT), we used the following Equations (1) and (2):

(1) $$PDL=\frac{log\frac{N_{f}}{N_{i}}}{log2}$$

(2) $$PDT=\frac{t}{PDL}$$

where PDL means the population doubling level, N_f_ is the final cell number at harvest, N_i_ is the initial number of cells seeded into the culture dish, and t is the time from seeding to harvesting [56,57,58].

Cells were seeded at the beginning of the experiment at a density of 50,000 cells per T-25 flask. Cells were passaged on the seventh day of the experiment and seeded again at 50,000 per T-25 flask (N_i_ = 50,000). After another seven days, i.e., on the fourteenth day of the experiment, the cells were trypsynized and counted. The cell number obtained was taken as N_f_, while the t value in the calculation was 168 h (7 days).

### 5.5. Homocysteine Level Analysis

The culture medium change in both melanoma lines took place on the third, fifth, seventh (passaging), tenth, and twelfth days of the experiment. On the fourteenth day of the experiment, cell culture media were collected, centrifuged and stored at −20 °C. The Homocysteine ELISA Kit (catalog No. abx513964, Abbexa, Cambridge, UK) was used according to the manufacturer’s protocol. Measurements were made using the Infinite 200 PRO microplate reader and Magellan software version 7.2 (both from Tecan, Grödig, Austria).

### 5.6. Cell Cycle Analysis

The cellular DNA content was measured by a NucleoCounter NC-3000 to reveal the cell distribution within the major phase of the cell cycle. For this purpose, cells were stained with the fluorescent dye DAPI, which attaches to double-stranded DNA, and the intensity of fluorescence is proportional to the DNA content. In brief, the cells were trypsinized, counted, and suspended in a mixture of Dulbecco’s Phosphate-Buffered Saline (Paisley, UK) and 70% ice-cold ethanol (Chempur, Piekary Śląskie, Polska). This suspension was then stored at 2–8 °C. Cell pellets were next incubated (5 min, 37 °C) with Solution 3 (ChemoMetec, Lillerød, Denmark), consisting of 1 μg/mL DAPI and 0.1% Triton X-100 in PBS. The samples were applied to NC-Slides A8 (ChemoMetec, Lillerød, Denmark), and cytometric analysis was performed.

### 5.7. Statistics

Statistical evaluation of the results was performed using GraphPad Prism 8.0 (GraphPad Software, San Diego, CA, USA). Normality of the data was checked by the Shapiro–Wilk test, and then homogeneity of variances was checked with the Brown–Forsythe test. The statistical significance of differences between groups was tested using one-way ANOVA followed by Dunnett’s or Tukey’s test.

## Data Availability

Data will be made available on request.

## Supplementary Materials

The following supporting information can be downloaded at https://www.mdpi.com/article/10.3390/ijms26041540/s1.
