# Supplementary material for: Novel Method for the Synthesis of Hydroxycobalamin[c-lactam] and Its Impact on Melanoma Cells In Vitro
Source: Int J Mol Sci. 2025 Feb 12;26(4):1540. doi: 10.3390/ijms26041540 (PMC11855847; doi:10.3390/ijms26041540)
Supplement: Supplementary file 1 [file ijms-26-01540-s001.zip › ijms-3405480-supplementary.pdf]

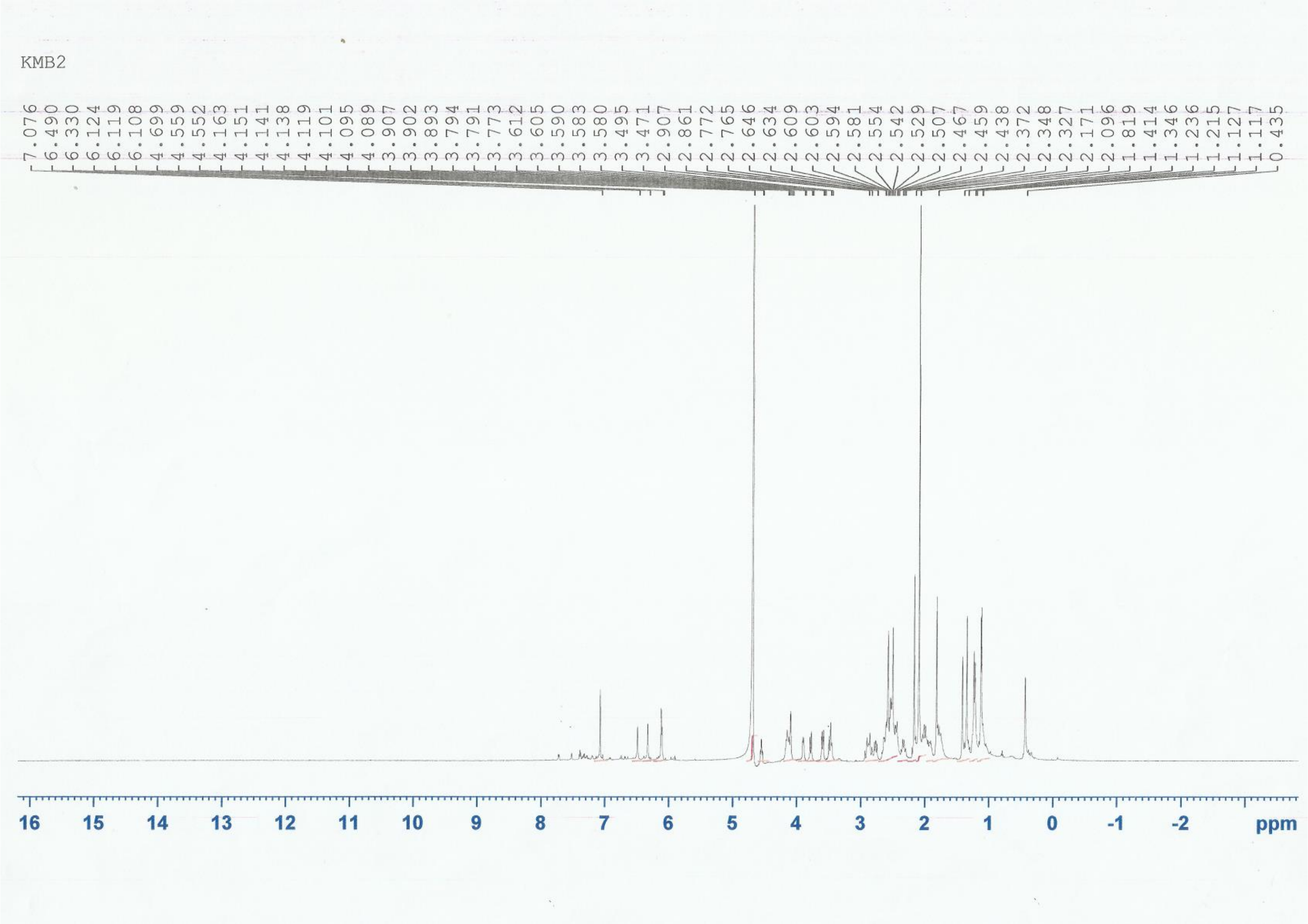

Figure S1. ^{1}H NMR spectra of hydroxycobalamin[*c*-lactam].

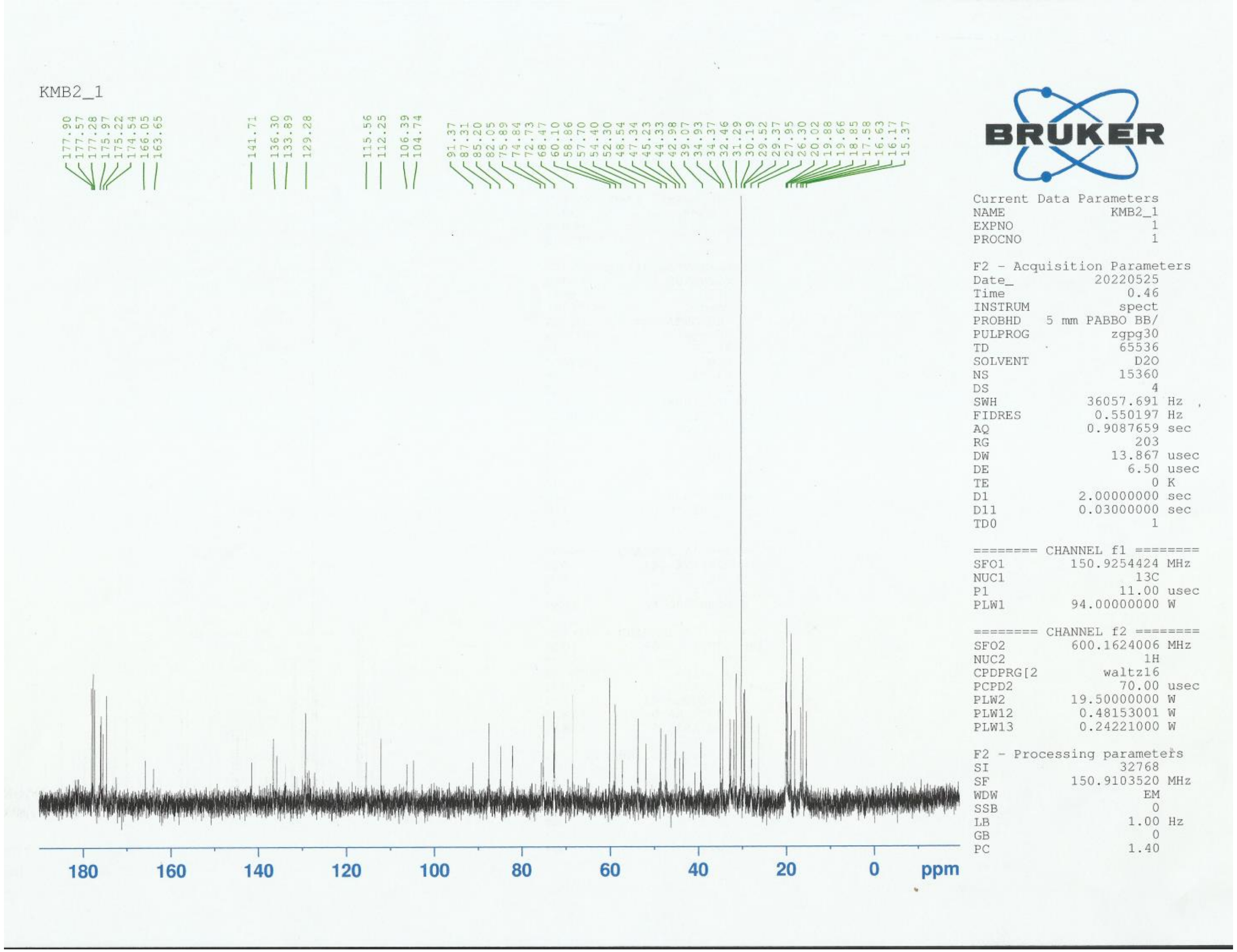

Figure S2. ^{13}C NMR spectra of hydroxycobalamin[*c*-lactam].

Compound Spectrum List Report

Analysis Info

Analysis Name	D:\Data\B3HCl.d	Acquisition Date	
Method	low_mass.m	Operator	KM
Sample Name	1-tolil	Instrument	impact II 1825265.10082
Comment			

Acquisition Parameter

Source Type	ESI	Ion Polarity	Positive	Set Nebulizer	0.3 Bar
Focus	Active	Set Capillary	4000 V	Set Dry Heater	200 °C
Scan Begin	100 m/z	Set End Plate Offset	-500 V	Set Dry Gas	3.0 l/min
Scan End	1400 m/z	Set Charging Voltage	2000 V	Set Divert Valve	Source
		Set Corona	0 nA	Set APCI Heater	0 °C

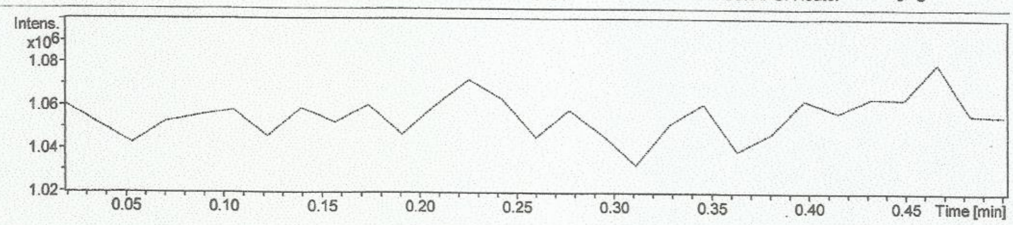

+MS, 0.0-0.5min #1-29

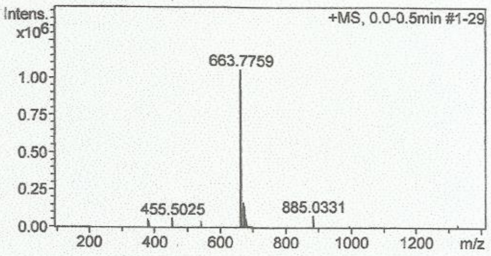

#	m/z	Res.	S/N	I	I %	FWHM
1	397.1388	15851	764.0	52282	5.0	0.0251
2	455.5025	14552	653.4	65139	6.2	0.0313
3	542.2279	16270	374.1	50307	4.8	0.0333
4	663.7759	23583	6635.9	1055199	100.0	0.0281
5	674.7671	16264	1078.7	172926	16.4	0.0415
6	885.0331	16139	374.2	82773	7.8	0.0548
7	995.6616	14984	53.2	11240	1.1	0.0664
8	1327.5195	12570	137.4	14136	1.3	0.1056

Figure S3. HR MS spectra of hydroxycobalamin[*c*-lactam].

Chromatogram

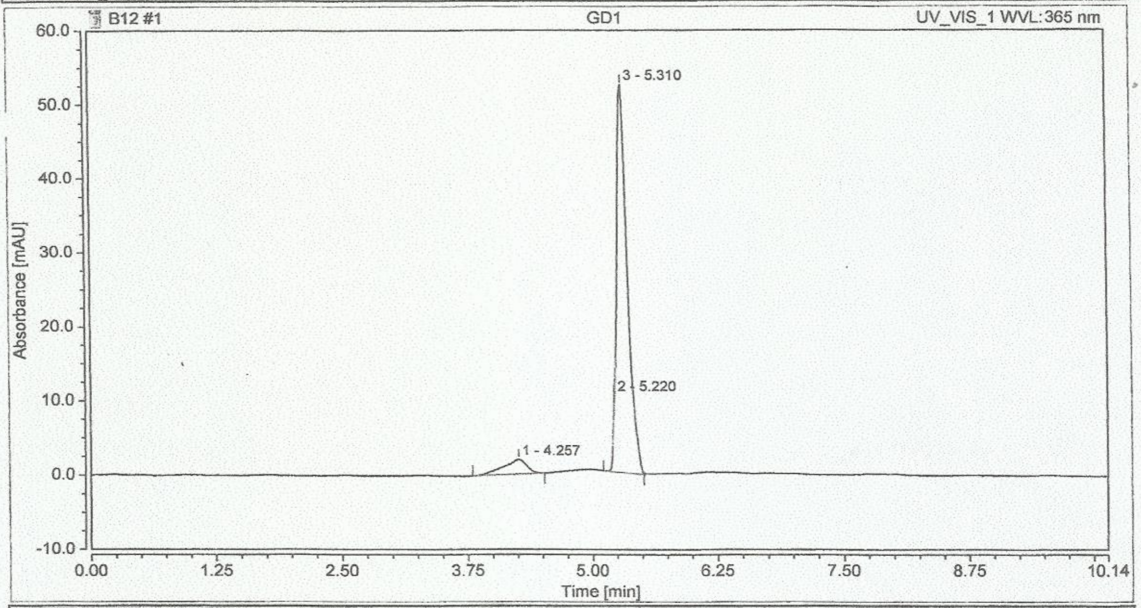

Integration Results

No.	Peak Name	Retention Time min	Area mAU*min	Height mAU	Relative Area %	Relative Height %	Amount n.a.
1		4.257	0.356	1.309	4.95	2.38	n.a.
2		5.220	0.023	1.297	0.32	2.36	n.a.
3		5.310	6.809	52.355	94.78	95.26	n.a.
Total:			7.188	54.961	100.00	100.00	

Default DAD/Integration

Chromeleon (c) Dionex
Version 7.2.2.6686

Figure S4. HPLC analysis of hydroxycobalamin[*c*-lactam].
